# Medical Education

**Published:** 1871-03

**Authors:** J. Henry Carstens


### Selections.

---

### MEDICAL EDUCATION.

BY J. HENRY CARSTENS, M. D.

Some of the medical journals, as well as some professors in their introductory addresses, have lately written and said very much about medical education, especially in reference to the value, comparatively, of clinical and didactic instructions. Some have tried to depreciate the usefulness of clinical teaching, saying that the student is fascinated by this kind of instruction, and impressed with the idea that by this means he can acquire more rapidly and easily, a perfect knowledge of the art and science of medicine, and that the student therefore neglects the primary studies, and devotes most of his time to the hospital and dispensary. There is some truth in this, undoubtedly, but like almost every other question, it has two sides. We do not think that a college can be censured more for paying special attention to the clinical advantages that it offers, than a college that prides itself on its thorough didactic instruction.

The fault of an insufficient education in medicine doubtless lies in the lack of the harmonious co-operation of the different medical institutions of this country. Why are all efforts to raise the standard of medical education failures? What must the impression be on the people at large, of this want of unison in the medical profession? With very few exceptions, the different colleges require three years of study, with two courses of lectures. Why not require three courses of lectures? The first year that is spent in a physician's office is often so much time lost; there are few practicing physicians who devote time to the student, and take pains to instruct him. But there are such noble men. We have known students who knew more after studying two or three years with such a man, than most graduates do. Any one, whatever may have been his previous history, can show a certificate that he has studied three years, and signed properly by an M. D.

But the principal aim of the profession should be to systematize the study of medicine. A first course student listens to all lectures, from anatomy to ophthalmology. It is almost impossible to study anatomy, and all the other branches of medicine, during one course of lectures, to any advantage—dissecting one hour, while the next is spent with an ophthalmoscope trying to detect minute changes that a retina has undergone. Still, at present, this is the rule in most colleges ; the junior student, as well as the senior, attends all the various lectures and also the clinics.

During the first course a student should only study a few, and those the fundamental, branches of medicine; anatomy, physiology, materia medica and chemistry. To these his whole time ought to be devoted. At the end of the term he should be examined, and if he passes he may proceed with the remaining branches; but if he fails he should again commence at the beginning. The second course should be devoted to didactic instruction in practice of medicine, surgery, obstetrics, etc. At the end of this term another examination, as before, should take place. The third course should be principally clinical: clinical surgery, practical midwifery, dermatology, practical instruction in the use of instruments, as the ophthalmoscope, laryngoscope, ete., auscultation and percussion. With such a course of instruction we think that a person with only ordinary reasoning faculty could safely be intrusted with the health and life of the community.

But even now, while only two courses of lectures are required, decided improvement can be made by having the class separated. In the first the fundamental branches are taught, and in the second course the practical branches, with clinical instructions. But without thorough clinical tuition no student ought to receive a diploma.

We ask those who try to depreciate the value of studying diseases at the bedside, if they would risk the lives and health of their children in the hands of physicians who acquire their knowledge from books alone. Take, for example, hip joint disease, which can only be distinguished by a practiced eye in its early stage, and this is the time to begin treatment, if you want to avoid its fearful results. But how few young physicians can take a diagnosis of this disease in its very commencement. Take disease of the eye, requiring ophthalmoscopic examination; who can make a correct diagnosis in his first effort? Then how difficult is it to distinguish skin diseases. Auscultation and percussion are only of value to him who has practiced on a diseased person as well as on a healthy one. How many eyes have been lost, how many patients sent to the grave by this lack of instruction on the part of the physician while a student.

It is certainly of no use for students to see many patients, and be merely told by their professors that one is a case of bronchitis and another typhoid fever; the students ought to be called upon in turn to make their *own* diagnosis, and then be thoroughly questioned in reference to differential symptoms, treatment, etc. By this means a student learns how to examine a patient; why, we have seen graduates who could not question their patients so that a correct diagnosis could be made. They ask one question about a certain organ or part, and then about another, and then again commence with the first, and so on.

But enough for the present. Let another effort be made; let the professors encourage the junior students to study only the primary branches, and at the end of the term pass an examination. If they pass satisfactorily, let the examination on these subjects be omitted at the end of the second course. By this system we think that a higher standard of medical education would be gained, and it would pave the way for the formation of classes, as above indicated.

